# Identification of Molecular Correlations Between DHRS4 and Progressive Neurodegeneration in Amyotrophic Lateral Sclerosis By Gene Co-Expression Network Analysis

**DOI:** 10.3389/fimmu.2022.874978

**Published:** 2022-04-11

**Authors:** Shu Li, Yu Zhu, Caihui Wei, Cheng Li, Wenzhi Chen, Shishi Jiang, Dongxiang Yuan, Renshi Xu

**Affiliations:** Department of Neurology, Jiangxi Provincial People’s Hospital, Affiliated People’s Hospital of Nanchang University, Nanchang, China

**Keywords:** amyotrophic lateral sclerosis, weighted gene co-expression network analysis, neurodegeneration, DHRS4, DHRS3

## Abstract

Amyotrophic lateral sclerosis (ALS) is a fatal neurodegenerative disease, and its candidate biomarkers have not yet been fully elucidated in previous studies. Therefore, with the present study, we aim to define and verify effective biomarkers of ALS by bioinformatics. Here, we employed differentially expressed gene (DEG) analysis, weighted gene co-expression network analysis (WGCNA), enrichment analysis, immune infiltration analysis, and protein–protein interaction (PPI) to identify biomarkers of ALS. To validate the biomarkers, we isolated the lumbar spinal cord from mice and characterized them using Western blotting and immunofluorescence. The results showed that Dhrs4 expression in the spinal cord was upregulated with the progression of SOD1^G93A^ mice, and the upregulation of DHRS4 and its synergistic DHRS3 might be primarily associated with the activation of the complement cascade in the immune system (C1QA, C1QB, C1QC, C3, and ITGB2), which might be a novel mechanism that induces spinal neurodegeneration in ALS. We propose that DHRS4 and its synergistic DHRS3 are promising molecular markers for detecting ALS progression.

## Introduction

Amyotrophic lateral sclerosis (ALS) is a chronic degenerative disease characterized by degeneration of the upper and lower motor neurons (1), and most patients with ALS eventually die due to respiratory failure, with an average survival time of about 3–5 years (2). An important pathological hallmark of ALS is the presence of neuronal cytoplasmic inclusions in degenerating motor neurons and oligodendrocytes. These inclusions are toxic to neurons, which are thought to accelerate the death of neurons and eventually develop to ALS. Currently, it is accepted that superoxide dismutase 1 (SOD1) and TAR DNA-binding protein 43 (TDP-43), fused in sarcoma/translocated in sarcoma (FUS) and chromosome 9 open reading frame 72 (C9ORF72), are the main contents of the neuronal inclusions (3, 4).

Neurodegenerative diseases, including Alzheimer’s disease (AD), Parkinson’s disease (PD), multiple sclerosis, Huntington’s disease, and ALS share some common features in pathogenesis and pathology. Endoplasmic reticulum stress, chronic neuroinflammation, autophagy, mitochondrial dysfunction, oxidative stress, and DNA damage are recognized as the major neuropathological hallmarks of neurodegenerative diseases (5–10). SOD1 is a crucial antioxidant enzyme responsible for scavenging free radicals (11). Therefore, a series of the mutated SOD1s modify the protein activity, causing the accumulation of toxic hydroxyl radicals and neurodegeneration (12). **T**he conformational changes of SOD1 are implicated to accelerate the aging process and also manifested in other aging-related neurodegenerative diseases like PD (13) and AD (14). SOD1, as the second most commonly identified cause of ALS, makes the SOD1 protein more prone to aggregation, resulting in the deposition of cellular inclusions that contain misfolded SOD1 aggregates. It is known that about 20% of familial ALS cases are strongly associated with mutated SOD1 (12, 15). However, mutations in SOD1 have also been linked to some sporadic ALS (16, 17). These results suggest a similar mechanism among SOD1 aggregates in ALS, PD, and AD, which could lead to the development of novel therapeutic targets for both neurodegenerative diseases. Despite numerous studies that have been published, mainly reported in the field of ALS, the underlying mechanisms by which SOD1 mutations cause neurodegeneration has not been fully understood. Currently, because the potential pathogenesis of ALS remains elusive, there are no effective therapies or prevention for ALS. Thus, it is crucial to explore novel molecular mechanisms of ALS pathogenesis, thereby identifying novel therapeutic strategies.

The rapid development of bioinformatics has shown great prospects in generating novel insights into disease mechanisms and diagnostic and therapeutic targets. Weighted gene co-expression network analysis (WGCNA) is an important method to find a correlation between gene sets and clinical traits (18) and can be used to identify candidate biomarkers. Differentially expressed gene (DEG) analysis is typically detected for differences in expression of transcriptomics data or microarray data, and it is widely used to explore potential biomarkers of diseases in the biomedical field. Therefore, using WGCNA can be useful for exploring the biomarkers of ALS, and research on molecular mechanisms may offer novel insight into the progression of ALS.

**Figure 1:**
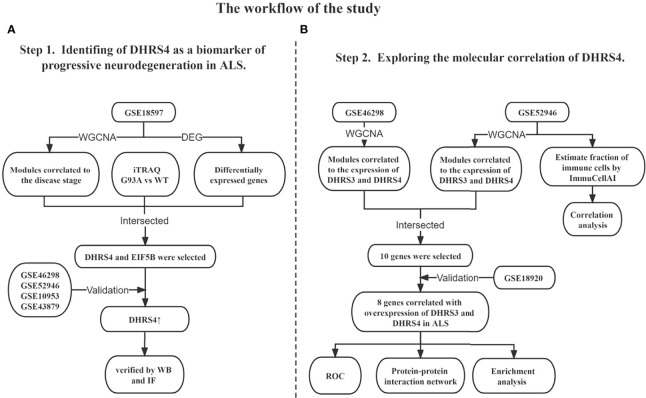
Overview of the study design. **(A)** Identification of DHRS4 as a biomarker of progressive neurodegeneration in ALS. **(B)** Exploration of the molecular correlation of DHRS4. WGCNA, weighted co-expression network analysis; DEG, differentially expressed gene; iTRAQ, isobaric tags for relative and absolute quantitation; WT, wild type; WB, Western blotting; IF, immunofluorescence; ROC, receiver operating characteristic; ALS, amyotrophic lateral sclerosis.

In this study, to our knowledge, for the first time, we found that dehydrogenase/reductase (SDR family) member 4 (DHRS4) was upregulated in the spinal cord with disease progression of ALS by performing bioinformatics analysis, and our preliminary experiments had also verified this view. In addition, dehydrogenase/reductase (SDR family) member 3 (DHRS3), also a member of the short-chain SDRs family, showed a similar expression trend. It is worth mentioning that the biological functions of DHRS3 and DHRS4 also have high overlap in that both catalyze the reduction of all-trans retinal to all-trans retinol in the presence of NADPH (19–22). Consequently, in this study, we included DHRS3 as a synergistic gene of DHRS4 together with further analysis. To further explore the potential mechanisms of DHRS3 and DHRS4 in ALS, we performed a comprehensive molecular association exploration. Hence, our study is expected to provide novel insights into the pathogenesis and progression of ALS and identify distinct biomarkers that correlate with neurodegeneration. The analysis procedure of our study is shown in **Figure 1**.

## Material and Methods

### Animals

The SOD1^G93A^ transgenic C57BL/6J mice and wild-type (WT) mice in our experiment were obtained from the Institute of Model Animals of Nanjing University. The SOD1^G93A^ mouse model of ALS is a viable and fertile transgenic expression of the human SOD1^G93A^ mutant form (23). The mice were under housing conditions with a 12 h light/dark cycle, 20°C–27°C, 40%–50% humidity, and free access to food or water. The DNA of mouse tail was extracted for SOD1 genotyping when mice were 4 weeks old by PCR as in our previous studies (24, 25). Mice used in the experimental operation and procedures were housed following the protocols approved by the Animal Management Committee of the Jiangxi Provincial People’s Hospital.

### Dataset Preparation

A total of 6 datasets, GSE18597, GSE52946, GSE10953, GSE43879, GSE46298, and GSE18920, were used in this study, and all of them were obtained from the Gene Expression Omnibus (GEO, http://www.ncbi.nlm.nih.gov/geo). GSE18597 (spinal cord samples from 21 SOD1^G93A^ mice and 21 WT mice) and GSE46298 (spinal cord samples from 16 129Sv_G93A mice, 16 129Sv_WT mice, 16 C57_G93A mice, and 16 C57_WT mice) were obtained using the platform Affymetrix Mouse Genome 430 2.0 Array; GSE52946 (lumbar cord samples from 10 ALS and 10 controls) was obtained from the platform Illumina HiSeq 2000 (*Homo sapiens*). The microarray datasets were pre-processed quantile normalized by limma package (26). GSE18597 was used to screen DEGs and for WGCNA. GSE52946, GSE10953, GSE43879, and GSE46298 were used for the validation of candidate genes. Meanwhile, GSE52946, GSE46298, and GSE18920 were further selected for functional analysis.

### Weighted Gene Co-Expression Network Construction

WGCNA was constructed using a WGCNA package to screen hub genes related to the ALS stage. First and foremost, the similarity matrix was constructed by using the expression data by calculating Spearman’s correlation coefficient. Next, the similarity matrix was transformed into a topological overlap matrix (TOM) and selected an appropriate soft threshold power to meet the criteria for constructing a scale-free network. 1-TOM was used as the distance to cluster the genes, and then the dynamic pruning tree was built to identify the modules. Similar modules were merged following a height cutoff of 0.25. The module showing the highest correlation with the ALS stage was selected for further analysis.

### Differential Gene Expression

The DEG analysis between SOD1^G93A^ and WT mice applied the limma package on the R platform, and the genes with an absolute log_2_ fold change greater than 0.5 and adjusted p-value less than 0.05 were used as cutoff values. Volcano plots were used to illustrate the differential expression of DEGs.

### Validation of the Hub Genes

To increase the reliability of our analysis, external databases were used to verify the interesting genes. Here, we carried out validation under four datasets, including GSE52946, GSE10953, GSE43879, and GSE46298.

### Evaluation of Immune Cell Infiltration

The ImmuCellAI (http://bioinfo.life.hust.edu.cn/web/ImmuCellAI/) tool (27) based on the expression data of microarray or RNA-Seq can accurately predict the abundance of 24 types of immune cells in the sample. To investigate the relationship between DHRS4 and immune cell infiltration, the expression file of GSE52946 was uploaded to ImmuCellAI to estimate the abundance of immune cells. Spearman’s correlation analysis was used to describe the correlation between DHRS4 and immune cells. p < 0.05 was considered statistically significant.

### Enrichment Analysis and Protein–Protein Interaction Network Analysis

To obtain further insights into the function of the genes in the module, we referred to the Metascape (http://metascape.org) (28) to perform the enrichment analysis. The tool displays the first 20 enriched terms as a bar graph. Module genes were uploaded to the STRING database (http://www.string-db.org/) (29), and then the protein–protein interaction (PPI) network was drawn through the STRING database. The target genes in the PPI network act as nodes, and the line from two nodes indicates relevant interactions.

### Western Blotting Analysis

According to the bioinformatics results, we have further validated Dhrs4 expression in SOD1^G93A^ animal models by Western blotting. The lumbar spinal cord tissues were lysed in radioimmunoprecipitation assay (RIPA) buffer (P0013B; Beyotime, Shanghai, China) containing phenylmethylsulfonyl fluoride (P0100; Solarbio, Beijing, China). After incubation on ice for 30 min, the lysate solution was centrifuged at 12,000 × *g* at 4°C for 10 min. The protein concentration in the supernatants was measured using the BCA Protein Assay kit (PC0020; Solarbio, Beijing, China). Ten micrograms of total protein was denatured at 100°C for 5 min, fractionated by a 10% sodium dodecyl sulfate–polyacrylamide gel, and then transferred onto a polyvinylidene fluoride membrane (Millipore Corp., Bedford, MA, USA). Afterward, the membranes were blocked using phosphate-buffered saline (PBS) containing 5% skim milk for 1 h at room temperature. Primary antibody and dilutions overnight at 4°C used were as follows: rabbit anti-DHRS4 (15279-1-AP; ProteinTech, Wuhan, China) at 1:4,000 and mouse anti-β-actin (TA-09; ZSGB-bio, Beijing, China) at 1:3,000. The horseradish peroxidase (HRP)-conjugated anti-rabbit (A0208; Beyotime, Shanghai, China) and anti-mouse (A0216; Beyotime, Shanghai, China) were employed at 1:10,000 dilution and 1:6,000 dilution for 2 h at room temperature, respectively. Protein bands were visualized using Super ECL Plus (S6009L; UElandy, Shanghai, China), and the detailed procedures were the same as our previous study (25, 30).pt?>

### Immunofluorescence Staining

The lumbar spinal cord at 120 days of age was used for immunofluorescence staining. Spinal cords were embedded in OCT, and 12-µm cryosections were cut using the Leica cryostat (Wetzlar, Germany) and then placed in a −20°C refrigerator for storage. Sections were removed from the −20°C refrigerator and left at room temperature for 30 min. After that, the sections were permeabilized with 0.3% Triton X-100 for 10 min, followed by washing 3 times with 0.01 M of PBS buffer for 5 min each time. After being blocked with 5% bovine serum albumin (BSA) for 1 h, rabbit anti-Dhrs4 (1:200, DF3986; Affinity Biosciences, Cincinnati, OH, USA) and mouse anti-NeuN (1:200, ab104224; Abcam, Cambridge, MA, USA) were used overnight at 4°C. Secondary antibodies dilutions were as follows: CoraLite594-Donkey anti-rabbit IgG(H+L) (SA00013-8; ProteinTech, Wuhan, China) 1:200 and CoraLite488-Donkey anti-mouse IgG(H+L) (SA00013-5; ProteinTech, Wuhan, China) 1:200 incubated for 2 h at room temperature. Next, the sections were washed 5 times for each 5 min using 0.3% Tween 20 in 0.01 M of PBS buffer. Then, nuclei were then stained with DAPI (G1012; Servicebio, Wuhan, China) for 10 min and rinsed for 5 min in 0.01 M of PBS 3 times. Finally, sections were visualized with a Nikon E800 fluorescent microscope with a spot digital camera (Diagnostic Instruments, Sterling Heights, MI, USA). DHRS4 fluorescence intensity per neuron used an integrated optical density value calculated by the Image-Pro Plus (Media Cybernetics, Rockville, MD, USA).

### Statistical Analysis

Statistical analysis and visualizations were performed in R studio (Rstudio Inc., Boston, MA, USA) using R version 4.0.2. The Xiantao web tools (https://www.xiantao.love/) were used for data visualization. The association between continuous variables was assessed using Spearman’s correlation coefficient. All numerical data were expressed as mean ± SD and Student’s t-test, or Wilcoxon signed-rank test was used to estimate the differences between the two groups. Two-Way ANOVA was used to compare two groups at different ages. Receiver operating characteristic (ROC) curves analysis was performed to determine the area under the curve (AUC), sensitivity, and specificity of genes by pROC package. p-Values <0.05 were statistically significant.

## Results

### Weighted Gene Co-expression Network Construction and Identification Key Module

**Figure 2:**
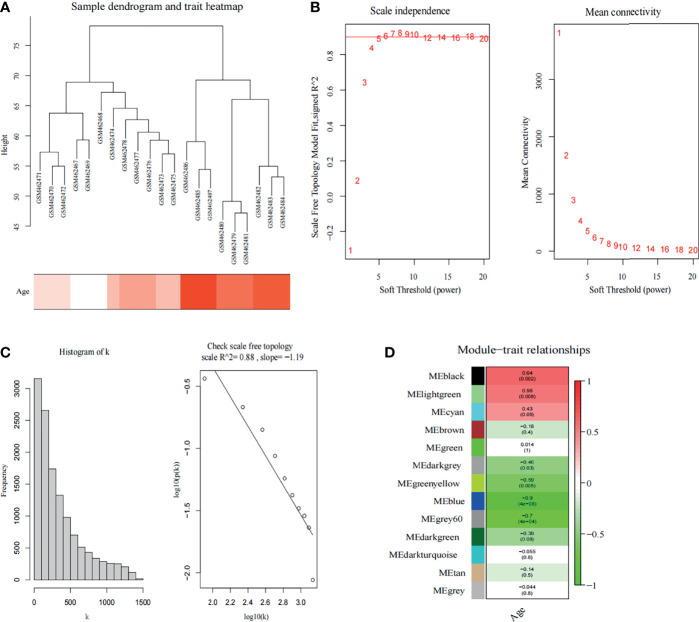
Weighted co-expression network analysis. **(A)** Sample dendrogram and trait heatmap. **(B)** Analysis of the scale-free fit index (left) and the mean connectivity (right) for various soft-thresholding powers. The red line indicates a correlation coefficient of 0.9. **(C)** The histogram of k and the correlation coefficient between k and p (k). **(D)** Heatmap shows correlations of module eigengenes with clinical traits. The numbers in each cell represent the correlation coefficients and p-values between clinical trait and module eigengenes.

A total of 21 SOD1^G93A^ mice samples were analyzed by co-expression analysis based on WGCNA. However, considering WGCNA is sensitive to the batch processing effect, we removed genes with zero variance among groups. After processing, a total of 12,990 genes were extracted for WGCNA. Then, the hclust function was used to confirm whether the data had outlier samples. The results revealed intragroup consistency, as well as the group differences, was clear; therefore all samples were included in the analysis (**Figure 2A**). To satisfy this condition to construct scale-free networks, we set the soft threshold power as 5, and by calculating the scale-free topology fitting index, the value of the R square reached 0.88 (**Figures 2B, C****)**. As shown in **Figure 2D**, a total of 10 non-gray modules were discovered. Among them, black module (correlation coefficient = 0.64, p = 0.002) and light-green modules (correlation coefficient = 0.56, p = 0.008) with an extremely strong positive correlation with the stage of SOD1^G93A^ mice.

### Selection and Validation of Hub Genes

**Figure 3:**
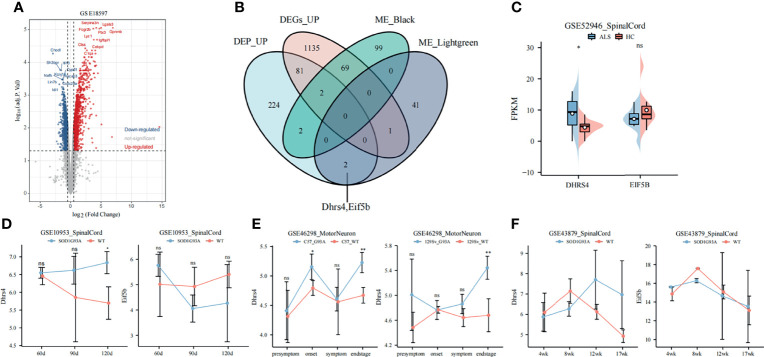
Selection and validation of hub genes. **(A)** The volcano plot of DEGs at 126 days of age in GSE18597. **(B)** The Venn diagram shows the intersection of DEGs, co-expression modules, and iTRAQ (130 days spinal cord) results. **(C)** The expression of hub genes in ALS patients and healthy controls. The DHRS4 expression is significantly upregulated in ALS patients. **(D)** Validation of hub genes in GSE10953. The Dhrs4 expression is significantly upregulated in SOD1^G93A^ mice compared with WT mice at 120 days. **(E)** Validation of Dhrs4 in GSE46298. The Dhrs4 expression is significantly upregulated in SOD1^G93A^ motor neuron at end stage. **(F)** Validation of hub genes in the GSE43879. The expression of Dhrs4 shows an increasing trend from early stage to end stage. *p < 0.05, **p < 0.01, ns, no significance. DEGs, differentially expressed gene; iTRAQ, isobaric tags for relative and absolute quantitation; ALS, amyotrophic lateral sclerosis; WT, wild type.

The expression values of 126-day samples in GSE18597 were used to identify DEGs. As shown in **Figure 3A**, 1,288 significantly upregulated genes and 1,310 significantly downregulated genes were identified. The highly connected genes of the black and light-green modules were investigated as potential key factors related to the stage of ALS. Thus, we selected the candidate hub genes from key modules. In addition, in our previous study, the isobaric tags for relative and absolute quantitation (iTRAQ) were applied to perform proteomic analysis on the spinal cord between SOD1^G93A^ mice and WT mice at 130 days of age (31). To screen stable and robust hub genes accurately, the selection of hub gene criteria is as follows: first of all, these genes were included in the key modules, with module membership >0.8 and gene significance >0.4; next, the genes presented in the progression stage of iTRAQ were upregulated; moreover, they are DEGs between WT mice and SOD1^G93A^ mice. Based on the above criteria, Eif5b and Dhrs4 were selected (**Figure 3B**).

The expression levels of EIF5B and DHRS4 were first validated in GSE52946. As shown in **Figure 3C**, the expression levels of DHRS4 were significantly higher in ALS spinal cord than in healthy controls, but EIF5B did not change significantly. In addition, in the spinal cord samples from GSE10953, the expression level of Dhrs4 upregulated in SOD1^G93A^ spinal cord at 120 days (**Figure 3D**). Similarly, as shown in **Figure 3E**, it was verified that Dhrs4 was upregulated in SOD1^G93A^ motor neurons at the end stage in GSE46298. Although the validation of Dhrs4 in GSE43879 was not statistically significant (**Figure 3F**), what cannot be ignored is that it shows an increasing trend. Furthermore, we found that in the various stages of ALS, EIF5B does not have a clear trend (**Figures 3C, D, F**). Based on preliminary analysis, we can see that DHRS4 has a conspicuous association with the symptomatic stage of ALS. We are very interested in that and select DHRS4 for subsequent analysis to study it further.

### The Expression of Dhrs4 Increased in the Neuron of Lumbar Spinal Cords of SOD1^G93A^ Mice at the Symptomatic Stage

**Figure 4:**
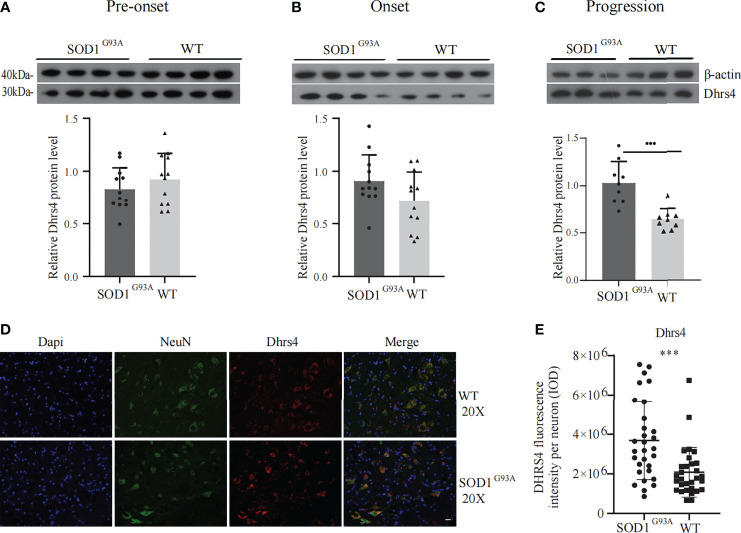
Validation of Dhrs4 by Western blotting and immunofluorescence. **(A–C)** Representative Western blotting images of Dhrs4 in SOD1^G93A^ mice compared with WT mice. **(A, B)** Dhrs4 expression has no significant difference in pre-onset and onset stages, respectively. **(C)** At progression stage of SOD1G93A mice, the expression of Dhrs4 is markedly upregulated. Data are presented as the mean ± SD (n = 3 or 4 per group), unpaired t-test, ***p < 0.001. **(D)** The expression of Dhrs4 in neurons is shown. The NeuN and Dhrs4 are double-labeled performed with anti-Dhrs4 (red) and anti-NeuN (green), respectively. **(E)** Quantifications of Dhrs4 fluorescence intensity per neuron (IOD). Data are presented as the mean ± SD (n = 3 per group), unpaired t-test with Welch’s correction; ***p < 0.001. Scale bars, 20 μm. WT, wild type. IOD, Integral optical density.

Subsequently, key hub genes were validated using Western blotting and immunofluorescence. The alteration of Dhrs4 expression in the lumbar spinal cords at the pre-onset (60–70 days), onset (90–100 days), and progression (120–130 days) stages of SOD1^G93A^ mice and the same period of WT mice was examined by Western blotting. The results illustrated that the expression of Dhrs4 in the lumbar spinal cord of progression stages of SOD1^G93A^ mice was significantly upregulated when compared to WT mice (**Figure 4C**). As indicated in **Figures 4A, B**, the expression of Dhrs4 did not change significantly between SOD1^G93A^ mice and WT mice at the stages of pre-onset and onset.

Immunostaining was performed in lumbar spinal cord sections obtained at 120 days of age from SOD1^G93A^ mice and WT mice. To examine the changes in the expression levels of Dhrs4 in neurons in SOD1^G93A^ mice, the NeuN and Dhrs4 double-labeled were performed. The cell morphology and distribution showed that Dhrs4 was mainly present in the cytoplasm of neurons. Compared with SOD1^G93A^ mice, the staining of Dhrs4 in the WT mice was less dense, which may indicate that the expression level of Dhrs4 in SOD1^G93A^ mice is higher than that in WT mice (**Figures 4D, E****)**. Together, these results are qualitatively consistent with the results of the bioinformatics analysis and suggest that Dhrs4 overexpression seems to be implicated in exacerbating ALS progression.

### Identifying Synergistic Genes of DHRS4

**Figure 5:**
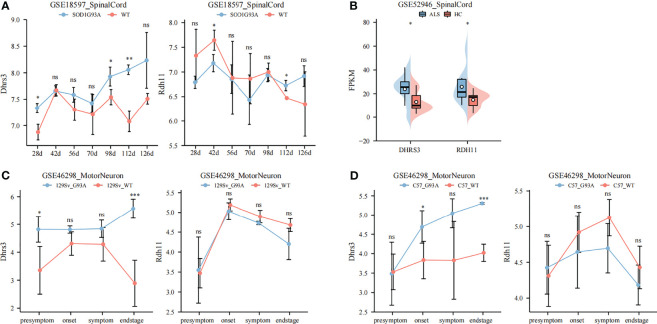
Identifying DHRS3 as a synergistic gene of DHRS4. **(A)** Validation of Dhrs3 and Rdh11 in GSE18597. The expression of Dhrs3 is significantly higher in SOD1^G93A^ mice. **(B)** Validation of Dhrs3 and Rdh11 in GSE52946. The expression of Dhrs3 and Rdh11 is upregulated significantly in ALS patients. **(C, D)** Validation of Dhrs3 and Rdh11 in GSE46298. The expression of Dhrs3 is significantly higher in SOD1 G93A mice. *p < 0.05, **p < 0.01, ***p < 0.001. ns, no significance; ALS, amyotrophic lateral sclerosis.

**Figure 6:**
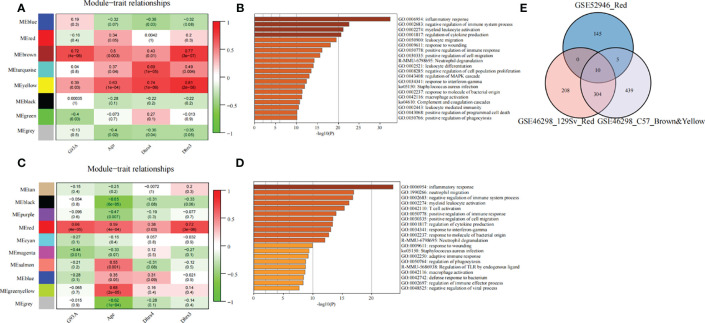
Exploring the molecular correlation of DHRS4. **(A)** Heatmap shows correlations of module eigengenes with age, Dhrs4, or Dhrs3 of SOD1^G93A^ mice (C57) in GSE46298. **(B)** Enrichment analysis of the key module. **(C)** Heatmap shows correlations of module eigengenes with age, Dhrs4, or Dhrs3 of SOD1^G93A^ mice (129Sv) in GSE46298. **(D)** Enrichment analysis of the key module. **(E)** Venn diagrams indicate 10 shared genes from key modules from GSE52946 and GSE46298.

The previous part of our study showed that the overexpression of DHRS4 was closely related to the progress of ALS, so it was necessary to further explore the mechanism of DHRS4. Because RDH11, DHRS3, and DHRS4 are important retinal reductases in the retinol metabolic pathway, changes of RDH11 and DHRS3 between SOD1^G93A^ mice and WT mice also were validated. The results showed that there was a significant upregulation of Dhrs3 expression in the spinal cord of progressive SOD1^G93A^ mice compared with WT mice (**Figures 5A–D**). It was also evident that the expression of Dhrs3 increased with disease progression. As shown in **Figures 5A–D**, there was no significant upregulation of Rdh11 expression in either SOD1^G93A^ mice or ALS patients. In the subsequent WGCNA analysis, the DHRS3 expression- and DHRS4 expression-related modules were identical (**Figures 6A, C****)**. DHRS3 and DHRS4 exhibited a similar trend of increase and shared similar biological functions, thus identifying DHRS3 as a synergistic gene of DHRS4.

### Exploring the Molecular Correlation of DHRS4

**Figure 7:**
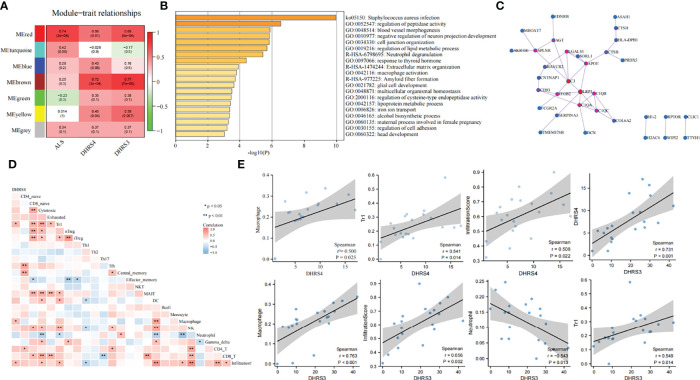
Function analysis of DHRS4 and DHRS3. **(A)** Building gene co-expression network of GSE52946 to identify gene modules that play important roles in conditions of DHRS4 or DHRS3. **(B)** Performing enrichment analysis in the red module. **(C)** PPI network of genes from the red module. The red nodes represent a central position. **(D)** Heatmap of infiltration immune cells associations with DHRS4. Blue color indicates r < 0, and red r > 0; *p < 0.05, **p < 0.01. **(E)** Spearman’s coefficient analysis of the relationship between immune cell infiltration level and DHRS4 or DHRS3. PPI, protein–protein interaction.

To reveal the mechanisms of the molecular function of DHRS4, we analyzed a series of datasets. First, co-expression network analysis was performed *via* WGCNA using the GSE52946. With Dhrs4 expression, Dhrs3 expression, and ALS patients as clinical traits, 7 non-gray modules were identified. In all of the co-expression network modules, the red module was positively correlated with three clinical traits (**Figure 7A**). As shown in **Figure 7B**, nerve cell development-related pathways, such as negative regulation of neuron projection development and glial cell development, as well as immune-related pathways, such as neutrophil degranulation and macrophage activation, are enriched in the red module. Additionally, enrichment analysis for the black module and light-green module of GSE18597 was performed. As depicted in **Supplementary Figure 1**, they were also enriched with immune-related pathways, such as pattern recognition receptor signaling pathway, lymphocyte anergy, and regulation of neutrophil extravasation, suggesting a potential role of the immune system in DHRS4 performing its function. The PPI network of the red module in GSE52946 shows that LRP1, C3, and C1QA/B/C were in a central position (**Figure 7C**).

Afterward, we also conducted an immune infiltration analysis of the GSE52946. Spearman’s coefficient analysis of the relationship between infiltrating immune cells and DHRS4 expression showed that macrophage (r = 0.500, p = 0.025), tr1 (r = 0.541, p = 0.014), and infiltration core (r = 0.508, p = 0.022) were positively correlated with DHRS4 expression (**Figures 7D, E****)**. Furthermore, we calculated the relationship between immune infiltration and DHRS3 expression. Of note, DHRS3 expression and DHRS4 expression have a high correlation (r = 0.731, p < 0.001) (**Figure 7E**). Not surprisingly, DHRS3 expression had high relevance for infiltrated immune cells. As shown in **Figure 7E**, the infiltration levels of neutrophils (r = −0.543, p = 0.013) were negatively correlated with DHRS3 expression; macrophage (r = 0.763, p < 0.001), tr1 (r = 0.549, p = 0.014), and infiltration core (r = 0.656, p = 0.002) were positively correlated with DHRS3 expression.

Later, WGCNA was also conducted on C57 and 129Sv mice from GSE46298. Nine non-gray co-expression modules used age, Dhrs4 expression, and Dhrs3 expression levels of mice as clinical traits (**Figures 6A, C****)**. Similarly, modules associated with clinical traits were selected for enrichment analysis. Genes in key modules of both 129Sv and C57 genotypes were mainly involved in immune-related pathways, such as inflammatory response, negative regulation of immune system process, positive regulation of immune response, neutrophil degranulation, macrophage activation, complement and coagulation cascades, and regulation of phagocytosis (**Figures 6B, D****)**. These results are consistent with the enrichment analysis of GSE52946 above.

### Validation and Evaluation of DHRS4-Associated Hub Genes

**Figure 8:**
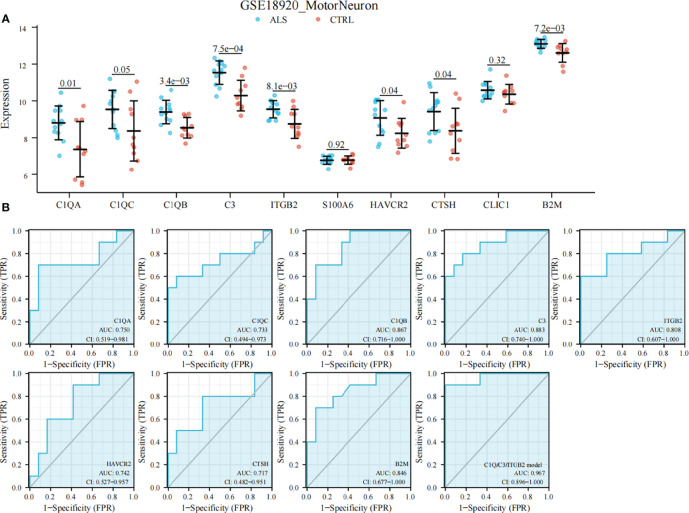
Validation of DHRS4-associated hub genes and ROC curve analysis. **(A)** Validation of hub genes in GSE18920. C1QA, C1QC, C1QB, C3, ITGB2, HAVCR2, CTSH, and B2M are significantly higher in ALS than controls. **(B)** ROC curve and AUC statistics to assess the diagnostic efficacy of DHRS4-associated hub genes for distinguishing between ALS patients and controls. Highest AUC value is seen for the C1Q/C3/ITGB2 model. ROC, receiver operating characteristic; ALS, amyotrophic lateral sclerosis; AUC, area under the curve.

**Figure 9:**
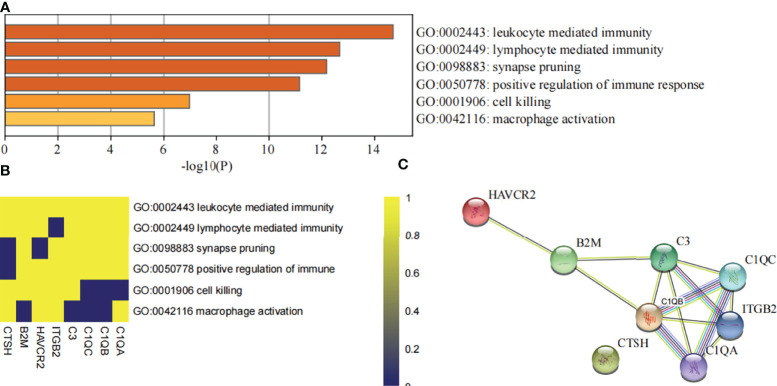
Evaluation of DHRS4-associated hub genes. **(A)** The bar charts show the top enriched pathways of DHRS4-associated hub genes. **(B)** Heatmap shows the association of DHRS4-associated hub genes and enriched signaling pathways. **(C)** PPI network analysis of DHRS4-associated hub genes. PPI, protein–protein interaction.

All of these findings suggest that DHRS4 may exert actions through the immune system. To investigate the specific mechanism of DHRS4 exerting its effect on ALS progression, we intersected the key module genes obtained in GSE52946 and GSE46298. A total of 10 genes were obtained (**Figure 6E**). Then, we validated the expression levels of these 10 genes in spinal motor neurons (sMNs) of GSE18920. As shown in **Figure 8A**, the expression levels of C1QA, C1QC, C1QB, C3, ITGB2, HAVCR2, CTSH, and B2M were significantly higher in ALS than in controls. Next, enrichment analysis revealed that the eight genes were mainly enriched in leukocyte-mediated immunity, lymphocyte-mediated immunity, synapse pruning, positive regulation of immune response, cell killing, and macrophage activation pathway (**Figures 9A, B****)**. In addition, we constructed the PPI network, and five hub genes (C3, C1QA, C1QB, C1QC, and ITGB2) were identified (**Figure 9C**). ROC curves showed that AUC values above 0.7 and even 0.9 indicated that the eight genes have diagnostic values (**Figure 8B**).

## Discussion

ALS is a fatal multifactorial neurodegenerative disease characterized by the selective death of motor neurons (32). In particular, the basis of selective vulnerability of sMNs in ALS has not been fully elucidated. SOD1^G93A^ transgenic mice are the most classical and widely used animal model in ALS research, with a survival period of approximately 4–5 months, mostly showing symptoms of progressive muscle weakness and atrophy at 90–120 days of age, which progressively worsen (23, 33, 34). As mentioned earlier in the text, in our previous study, protein quantification and differential analysis of the spinal cord of SOD1^G93A^ mice in the symptomatic stage (130 days of age) were performed by the iTRAQ, but the temporal relationship between the differential proteins and disease progression was not clarified (31). To clarify proteins closely related to the progression of ALS, in this study, WGCNA was used to identify and validate that Dhrs4 expression in the spinal cord was upregulated with the progression of SOD1^G93A^ mice. This suggests that overexpression of DHRS4 in the spinal cord may be a risk factor for the progression of ALS. DHRS4 expression was detected to be upregulated in neurons of the spinal anterior horn by immunofluorescence experiments; therefore, the exploration of molecular patterns associated with DHRS4 in sMNs is necessary. Also, DHRS3, which has a similar function to DHRS4, has a similar expression trend in the spinal cord of ALS mice and patients. WGCNA by combining human and mouse spinal cord transcriptome data identified eight associated genes that correlated with the expression of DHRS3 and DHRS4, which is particularly notable for the complement system characterized by C3/C1Q. This is also highly suggestive that DHRS3 and DHRS4 may influence the progression of ALS through the complement system.

As important retinal reductase in the retinol metabolic pathway, RDH11, DHRS3, and DHRS4 are mainly involved in the reduction of retinal into retinol and are also important regulatory enzymes in the all-trans retinoic acid (ATRA) synthesis pathway (20, 35, 36). Interestingly, Rdh11 is not significantly upregulated in sMNs of SOD1^G93A^ mice, even though it is also one of the retinal reductases. Regulation of DHRS3 or DHRS4 expression induces alterations in the synthesis of ATRA (37). Knockout of Dhrs3 results in a significant increase in ATRA and a significant decrease in retinol and retinal esters in mouse embryos. Dhrs3 knockout mice typically show embryonic death in late gestation, mainly from defects in heart development, indicating that Dhrs3 contributes crucially to controlling ATRA concentrations *in vivo* (38). Upregulation of the expression of DHRS3 and DHRS4 in sMNs implicates that more of the retinal is reduced to retinol, while less ATRA is irreversibly converted from retinaldehyde. In a previous study, we also found that Aldh1a2, the rate-limiting enzyme for ATRA synthesis, was downregulated in the spinal cord of SOD1^G93A^ mice and that Aldh1a2 expression was negatively correlated with neuronal death in the spinal cord (24). Furthermore, long-term ATRA administration to SOD1^G93A^ mice exerted neuroprotective effects by rescuing impaired retinoic acid signaling (39). These preliminary results support the hypothesis that insufficient ATRA synthesis in the spinal cord leads to underactivation of retinoic acid signaling and thus exacerbates disease progression and that upregulation of DHRS3 and DHRS4 appears to be the culprit responsible for these negative effects.

On the other hand, human DHRS4 has been reported to have carbonyl reductase activity, which can catalyze the reduction of 3-keto-C19/C21-steroids into corresponding 3β-hydroxysteroids, and inhibited by kaempferol, quercetin, genistein, and myristic acid (40–42). Interestingly, all of these pharmacological agents showed potential neuroprotective effects against ALS, especially in the SOD1 transgenic mouse model (43–47). This provides more reason to believe that DHRS4, and even DHRS3, can be used as potential targets for disease modification in ALS. Furthermore, in addition to inhibiting enzyme activity, the use of RNA interference or gene-editing techniques to inhibit gene expression is also an effective translational approach.

WGCNA revealed that the red module was closely associated with the upregulation of DHRS3 and DHRS4 in the lumbar medulla of ALS. Enrichment analysis revealed that the red module genes were associated with biological processes such as immune imbalance (especially C3/C1Q complement cascade, neutrophil degranulation, and macrophage activation), regulation of peptidase activity, and neurodevelopment. The PPI network revealed LRP1 and C1QA/B/C as hub genes in the module, while C3 degree was second only to LRP1. This is highly suggestive that the regulation of DHRS3 and DHRS4 may influence biological functions such as immune imbalance, peptidase activity, and neurodevelopment in the ALS spinal cord. Immune infiltration analysis revealed that the expression of DHRS3 and DHRS4 was significantly and positively correlated with type I regulatory T cells, macrophages, and infiltration score. In addition, DHRS3 expression was significantly correlated with dendritic cells, neutrophils, and CD4+ T cells. Although the correlation of DHRS4 with the above three cells was not significant, it had the same trend of correlation with DHRS3. This is also highly suggestive of a close association of the expression of DHRS3 and DHRS4 with at least the infiltration of tr1 cells, neutrophils, and macrophages, and this association was corroborated by the results of enrichment analysis, e.g., neutrophil degranulation and macrophage activation. It has been reported that ATRA enhances the phagocytosis of macrophages and is beneficial for immune modification. It has been reported that ATRA enhances the phagocytosis of macrophages and is beneficial for immune modification (48). Moreover, ATRA improves prognosis in a mouse model of acute ischemic stroke by modulating neutrophil function, inhibiting the formation of neutrophil extracellular traps, and increasing neutrophil phagocytosis by macrophages in the brain (49). The upregulation of DHRS3 and DHRS4 is bound to cause reduced ATRA synthesis, weaken the phagocytosis of macrophages, and cause difficulty in preventing the excessive infiltration and polarization of neutrophils, thus promoting the occurrence of immune imbalance in the spinal cord of ALS.

Given the significant upregulation of the expression of DHRS3 and DHRS4 in sMNs, WGCNA was used to explore gene modules closely associated with the expression of DHRS3 and DHRS4. Similar to the spinal cord transcriptome results in ALS patients, genes related to the immune system and neurodevelopment were closely associated with the expression of DHRS3 and DHRS4. Ten associated genes that significantly correlated with the expression of DHRS3 and DHRS4 were obtained. In the validation of GSE18920, eight associated genes (C1QA, C1QC, C1QB, C3, ITGB2, HAVCR2, CTSH, and B2M) were significantly upregulated in sMNs of ALS patients, and all of them showed favorable diagnostic values. With the exception of HAVCR2, seven of the eight genes have been previously reported to have upregulated expression in ALS studies. C1Q is the initiating component of the classical complement pathway formed by the α-, β-, and γ-chains (encoded by C1QA, C1QB, and C1QC genes, respectively), while C3 is central to both the classical and alternative complement pathways (50, 51). C1Q and C3 can be produced in the central nervous system by specific populations of neurons, microglia, and astrocytes (52, 53). Several studies have demonstrated induced C1Q and C3 accumulation in sMNs of ALS patients and that C1Q and C3 accumulation is strongly associated with disease progression in TDP43 transgenic mice (54–56). ITGB2 is upregulated in the blood of ALS patients (57), and ITGB2 expression in peripheral lymphocytes has the ability to identify prognosis in ALS patients (58). Upregulation of CTSH has also been reported in the spinal cord of SOD1^G93A^ mice but has not been studied thoroughly (59). Similarly, upregulated B2M has been reported in the peripheral blood of ALS patients (60, 61). ITGB2, C3, C1QA, C1QB, and C1QC were observed as central genes in PPI analysis, and each of them performed an important role in synaptic pruning. C1Q and C3 (initial activation components of the complement cascade) have been shown to have critical beneficial roles in the refinement of synaptic circuits during developmental stages and adult plasticity. However, excessive synaptic pruning in the adult nervous system may be detrimental and is associated with pathological synaptic loss (62). There is no doubt that the development of synaptotoxics induced by C1Q and C3 is equally present in ALS (63). Interestingly, retinoic acid signaling activated by ATRA significantly induces synaptic plasticity in neurons of multiple brain regions of the adult brain (64–66). In this study, we found that the expression of DHRS3 and DHRS4 in sMNs was closely associated with ITGB2, C3, and C1Q, key genes of synaptic pruning. Therefore, it can be hypothesized that upregulated DHRS3 and DHRS4 lead to reduced ATRA synthesis and lack of regulation of synaptic plasticity to cope with the occurrence of excessive synaptic pruning induced by co-expressed C1Q and C3; this process might contribute to neurodegeneration. This study explores and validates, based on our previous results, that DHRS4 and synergistically altered DHRS3 are closely associated with sMN degeneration in ALS. It was preliminarily clarified that the aberrant retinoic acid signaling in the ALS spinal cord may be derived from the deficient ATRA synthesis due to the upregulated DHRS3 and DHRS4. A possible mechanism for the C1Q/C3 complement cascade to promote neurodegeneration was proposed by identifying DHRS3- and DHRS4-related genes. Certainly, the results of this study are mainly from bioinformatics analysis, and further experiments are needed to confirm them.

In conclusion, the overexpression of DHRS4, and its synergistic DHRS3, may be primarily associated with the activation of the complement cascade in the immune system (C1QA, C1QB, C1QC, C3, and ITGB2), which may be a novel mechanism that induces spinal neurodegeneration in ALS.

## Data Availability Statement

Publicly available datasets were analyzed in this study. These data can be found here: a total of 6 datasets, GSE18597, GSE52946, GSE10953, GSE43879, GSE46298, and GSE18920, were used in this study, and all of them were obtained from the Gene Expression Omnibus (GEO, http://www.ncbi.nlm.nih.gov/geo).

## Ethics Statement

The animal study was reviewed and approved by the Animal Management Committee of the Jiangxi Provincial People’s Hospital.

## Author Contributions

SL, YZ, and RX designed and conducted the study. SL, YZ, CW, CL, and SJ collected the data. SL, YZ, CW, and DY performed experiments. SL, YZ, CW, CL, SJ, DY, and RX performed the statistical analysis and interpreted the data. SL, YZ, CW, and RX wrote the manuscript. SL, YZ, and CW contributed equally to this work. All authors contributed to the final version of the manuscript and approved the final manuscript.

## Funding

The work was supported financially by grants from the National Natural Science Foundation of China (30560042, 81160161, 81360198, and 82160255), Jiangxi Provincial Department of Science and Technology (20192BAB205043), Jiangxi Provincial Department of Science and Technology Gan Po Elite 555 (Jiangxi Finance Elite Education Refers to [2015] 108), the Innovation Fund Designated for Graduate Students of Nanchang University (CX2018214), and the Innovation Fund Designated for Graduate Students of Jiangxi Province (YC2020-B038).

## Conflict of Interest

The authors declare that the research was conducted in the absence of any commercial or financial relationships that could be construed as a potential conflict of interest.

## Publisher’s Note

All claims expressed in this article are solely those of the authors and do not necessarily represent those of their affiliated organizations, or those of the publisher, the editors and the reviewers. Any product that may be evaluated in this article, or claim that may be made by its manufacturer, is not guaranteed or endorsed by the publisher.
