# Supplementary figures and images for: Identification of Molecular Correlations Between DHRS4 and Progressive Neurodegeneration in Amyotrophic Lateral Sclerosis By Gene Co-Expression Network Analysis
Source: Front Immunol. 2022 Apr 11;13:874978. doi: 10.3389/fimmu.2022.874978 (PMC9035787; doi:10.3389/fimmu.2022.874978)

**Figure S1.**Enrichment analysis of black module (A) and lightgreen module (B) in GSE19597.

**
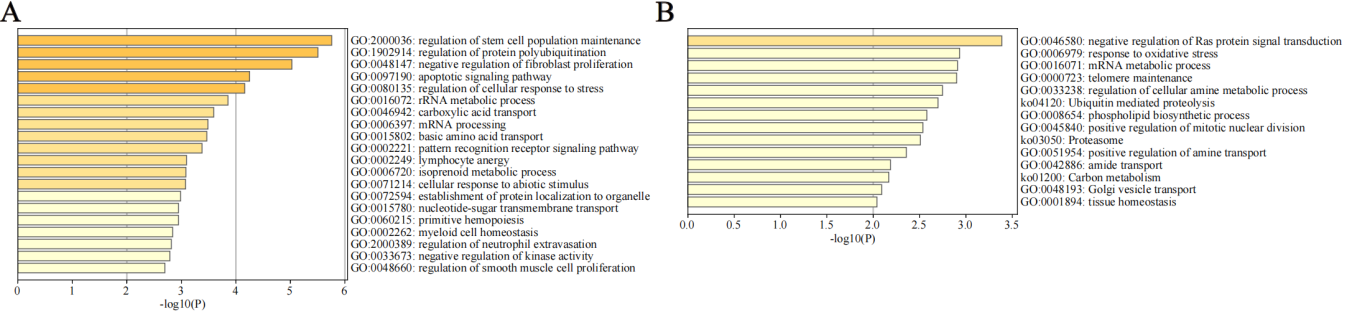
**

Supplement: Supplementary file 1 [file DataSheet_1.docx]
